# Elements of the Principles and Practice of Midwifery

**Published:** 1848-04

**Authors:** 


Art. VIII.—*Elements of the Principles and Practice of Midwifery.* By David H. Tucker, M.D., Professor of the Principles and Practice of Medicine, and formerly of Obstetrics and the Diseases of Women and Children, in the Franklin Medical College of Philadelphia. With numerous Illustrations. Philadelphia: Lindsay and Blakiston: 1848. 412 pp. 12mo.

This neat volume is one of a series, intended to comprise a library for practitioners and students. It is of portable size, well printed, on white paper, and strikes the eye favorably. The illustrations are well executed, and of a character to aid the student in comprehending the text. In a word, the work is just such an one as the practitioner would desire for a *vade mecum*, or the student for an introduction to the study of midwifery.

The anatomy of the female is presented in the first chapter, and this is followed by an account of the functions peculiar to the sex. A chapter is devoted to generation and the mode in which it is effected. The prevalent theory is summed up as follows:

"The female furnishes the germ, egg, or ovule; which pre-exists, or is produced, in iddefinite numbers, in the ovarium; which maturates at certain periods, ruptures its natural covering, and is brought into contact with the seminal fluid, either at the ovary, or in the oviduct, or in the uterus. The male, on the contrary, furnishes a fluid, composed not of animalcules, but of bodies having the power of motion, like the epithelial cells, &c.; that these bodies are carried by an unknown power to the fallopian tubes, or even to the ovaries; when, meeting with the ovule divested of its coverings, by a union of the two, how, we know not, *vitality results;* the *being* thus formed, being composed of principles from each parent, will at times present traits of resemblance, both moral and physical, to either one or the other, or perhaps both parents."

The ovum, fetal membranes, circulation and parts, are next described, to which succeeds a chapter on pregnancy, in all its forms, normal and abnormal, its signs and term of duration. The diseases incident to pregnancy are then briefly adverted to, and finally labor and its concomitant dangers complete the range of subjects comprised in the volume.

